# *n*-3 Polyunsaturated Fatty Acids and Mechanisms to Mitigate Inflammatory Paracrine Signaling in Obesity-Associated Breast Cancer

**DOI:** 10.3390/nu6114760

**Published:** 2014-10-30

**Authors:** Jennifer M. Monk, Harmony F. Turk, Danyelle M. Liddle, Anna A. De Boer, Krista A. Power, David W.L. Ma, Lindsay E. Robinson

**Affiliations:** 1 Department of Human Health and Nutritional Sciences, University of Guelph, Guelph, ON N1G 2W1, Canada; E-Mails: jmonk02@uoguelph.ca (J.M.M.); dliddle@uoguelph.ca (D.M.L.); adeboer@uoguelph.ca (A.A.D.B.); krista.power@agr.gc.ca (K.A.P.); davidma@uoguelph.ca (D.W.L.M.); 2 Guelph Food Research Centre, Agriculture and Agri-Food Canada, Guelph, ON N1G 5C9, Canada; 3 Institut Curie, Paris 75248, France; E-Mail: harmony.turk@curie.fr

**Keywords:** breast cancer, inflammation, obesity, adipokines, *n*-3 polyunsaturated fatty acids, leptin, adiponectin, aromatase, lipid rafts, eicosanoids

## Abstract

Globally, the prevalence of obesity is increasing which subsequently increases the risk of the development of obesity-related chronic diseases. Low-grade chronic inflammation and dysregulated adipose tissue inflammatory mediator/adipokine secretion are well-established in obesity, and these factors increase the risk of developing inflammation-associated cancer. Breast cancer is of particular interest given that increased inflammation within the subcutaneous mammary adipose tissue depot can alter the local tissue inflammatory microenvironment such that it resembles that of obese visceral adipose tissue. Therefore, in obese women with breast cancer, increased inflammatory mediators both locally and systemically can perpetuate inflammation-associated pro-carcinogenic signaling pathways, thereby increasing disease severity. Herein, we discuss some of these inflammation-associated pro-carcinogenic mechanisms of the combined obese breast cancer phenotype and offer evidence that dietary long chain *n*-3 polyunsaturated fatty acids (PUFA) may have utility in mitigating the severity of obesity-associated inflammation and breast cancer.

## 1. Introduction

Based on body mass index (BMI), globally 1.5 billion people are overweight (BMI ≥ 25.0 kg/m^2^), and 500 million of these individuals are classified as obese (BMI ≥ 30.0 kg/m^2^) [1]. The clinical consequence of obesity is that it acts as an independent risk factor for several other pathologies, including cancer [1,2]. In this context, obesity is associated with increased mortality in several types of cancer, including breast cancer (BC) [3]. It is estimated that obesity contributes to 50% of all BC cases in older women [4]. Furthermore, considering the prevalence of obesity in younger populations and the projected expansion of the obese population [1], the impact on BC incidence is likely to be exacerbated in the future. Obesity increases the risk of developing the most common BC subtype, estrogen receptor (ER)-positive and progesterone receptor (PR)-positive BC (*i.e.*, hormone-sensitive form of the disease) [4,5,6,7]. Paradoxically the incidence of hormone sensitive BC increases with age, which coincides with increasing adiposity and decreasing circulating estrogen levels [8]. In fact, in postmenopausal women the majority of BC cases associated with obesity are ER-positive with a phenotype exhibiting larger and faster growing tumors that metastasize to axillary lymph nodes [4,5,9,10,11]. The positive association between BC development and obesity in postmenopausal women is well-established using multiple anthropometric indices of obesity including BMI, adiposity and waist:hip circumference ratio [4,5,9,10,11,12]. Interestingly, postmenopausal BC risk is increased by the degree of weight gain during adult life prior to menopause [13,14], thereby indicating that the effects of obesity during the premenopausal phase impact BC risk later in life. Conversely, in premenopausal women the link between BC risk and BMI as a measure of obesity status is more controversial [15,16,17]. Studies finding no association commonly normalize data to BMI alone, which is a poor discriminator of body fat and lean mass, and fails to account for visceral adiposity which is believed to be a more deleterious adipose depot compared to subcutaneous [18,19]. Premenopausal women with high BMI have been shown to develop significantly larger tumors and worse histopathological features including increased tumor vascularization and metastasis to axillary lymph nodes compared to healthy BMI BC patients [9]. Additionally, premenopausal obesity has been shown to increase the risk of developing triple negative BC (ER, PR and HER negative) [9,20] and hormone receptor-negative BC (ER and PR-negative) [21,22]. Collectively, these data indicate that independent of hormonal status, obesity increases overall BC risk, an effect that is, at least in part, attributed to inflammatory mechanisms and paracrine interactions (*i.e.*, cross-talk) between cell types within the mammary tissue that promote tumorigenesis [4,23,24].

The connection between obesity and ER-positive BC in post-menopausal women is likely attributable to two main interrelated factors that will be a key focus of this review, including: (i) increased adipose tissue (AT) mass and the associated increase in inflammatory mediator production (both locally and systemically); and (ii) elevated AT aromatase activation, which is up-regulated by inflammatory mediators and drives aberrant estrogen production within the AT, thereby promoting BC tumorigenesis. The obesity-associated inflammatory mammary tumor microenvironment is complex and the resultant phenotype is underscored by autocrine and paracrine interactions between adipocytes, tumor infiltrating macrophages (TAM) and epithelial cells, which produce AT-derived inflammatory mediators, collectively referred to as adipokines, which will be discussed in more detail herein. The inflammatory mammary tumor microenvironment should not be confused with “inflammatory breast cancer” (IBC), a rare (1%–6% of all breast malignancies) aggressive BC subtype with higher grade metastatic hormone receptor negative tumors that has been reviewed elsewhere [25,26]. Moreover, we provide evidence that dietary long-chain (LC) *n*-3 polyunsaturated fatty acids (PUFA), particularly fish oil (marine)-derived eicosapentaenoic acid (EPA, 20:5*n*-3) and docosahexaenoic acid (DHA, 22:6*n*3), which have well established anti-inflammatory effects in obesity [27,28,29,30,31,32,33,34,35,36,37,38,39,40,41] and anti-carcinogenic effects in BC [42,43,44,45,46,47,48,49,50,51,52,53,54,55,56], may represent an effective complementary approach in the prevention and/or treatment of obesity-associated BC by attenuating inflammatory adipokine-mediated paracrine interactions within the mammary tumor microenvironment.

## 2. Obese Inflammatory Phenotype

AT is an endocrine organ that secretes greater than 50 recognized proteins including several cytokines and chemokines, both of which are included in the term adipokine (*i.e.*, of AT origin) [57]. In the classic obese phenotype (reviewed elsewhere [2,58,59]), the tissue stress and remodeling that occurs in expanding visceral AT is associated with dysregulated adipokine secretion and a subsequent state of chronic, sub-clinical, low-grade, systemic inflammation [2,60]. The cellular source of these inflammatory mediators includes adipocytes and cells of the stromal vascular fraction (SVF) including endothelial cells, fibroblasts, macrophages and T cells [60]. These adipokines can influence whole-body metabolism, insulin sensitivity and inflammation through autocrine, paracrine and endocrine signaling. Most notably, in obesity both the local AT and circulating levels of inflammatory mediators, such as TNFα, IL-6, IL-1β, MCP-1, leptin and many others (reviewed by [58,59]), are elevated, while levels of adiponectin, an anti-inflammatory adipokine, are decreased [61]. Many of these same adipokines are up-regulated in obese BC and activate signaling pathways that drive inflammation-associated malignant transformation, and therefore, when present in the mammary tissue result in a more severe BC phenotype, as discussed in detail below.

## 3. *n*-3 Polyunsaturated Fatty Acids and Obesity

In obesity, marine source LC *n*-3 PUFA have been shown to modulate and improve several critical aspects of the obese phenotype, collectively reducing AT inflammation. Specifically, *n*-3 PUFA modulate the production of AT-derived adipokines by increasing anti-inflammatory adiponectin levels [27,28,29,30,31,32,33,34,35], while decreasing production of inflammatory mediators such as leptin [36,37,38,39] and cytokines including TNFα, IL-6 and MCP-1 [29,35,40,41]. Moreover, dietary *n*-3 PUFA have been found to reverse and/or improved obesity-associated hepatic steatosis and impairments in glucose metabolism and insulin sensitivity [27,28,29,35,62,63,64]. Collectively, these anti-inflammatory effects of *n*-3 PUFA alter the obesity-associated inflammatory microenvironment and improve the overall obese phenotype. One well-documented effect of *n*-3 PUFA is the suppression of inflammation by interfering with pro-inflammatory signaling cascades via peroxisome proliferator-activated receptor (PPAR)γ-dependent and independent mechanisms that involve up-regulation of adiponectin, in murine [65] and human adipocytes [66]. Additionally, PPARγ is involved in trans-repression of nuclear factor kappa-light-chain-enhancer of activated B cells (NFκB) transcriptional activity leading to decreased expression of NFκB responsive genes including several inflammatory cytokines (TNFα, IL-1β, IL-6 and MCP-1) [67]. In this connection, *n*-3 PUFA functioning as PPAR-receptor ligands also interfere with other transcription factors involved in inflammatory signal transduction pathways including AP-1, STAT-1 and NFAT [68].

*n*-3 PUFA can also perturb inflammatory signaling in obesity through PPARγ independent signaling mechanisms, most notably by acting as ligands for the G-protein coupled receptor 120 (GPR120) [69]. GPR120 has been shown to be partly responsible for the anti-inflammatory effects of DHA by using the adaptor β-arrestin2 to interfere with inflammatory mediator-stimulated NFκB activation in macrophages [69]. Additionally, EPA and DHA exert anti-inflammatory effects following their selective incorporation into the phospholipid fraction of cell membranes where they can act to decrease the signaling efficiency of protein complexes in lipid rafts [70], or serve as substrates for the synthesis of anti-inflammatory bioactive lipid mediators (*i.e.*, eicosanoids) [71,72]. Taken together, *n*-3 PUFA may beneficially modulate obesity-associated pro-inflammatory paracrine interactions between the different cell types within AT. Overall, *n*-3 PUFA utilize multiple mechanisms to suppress inflammatory signaling, thereby modulating the obesity-associated inflammatory phenotype.

## 4. *n*-3 Polyunsaturated Fatty Acids and Breast Cancer

**Figure 1:**
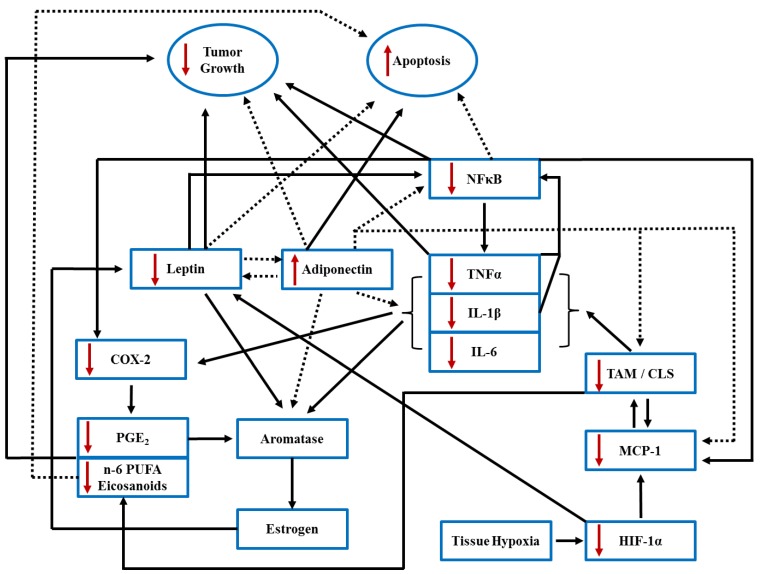
Summary of inflammatory mediator paracrine interactions produced within the obese mammary tissue tumor microenvironment highlighting the complex interactions mediated by adipocytes, macrophages and epithelial cells (main cellular sources of inflammatory mediators). Solid arrows denote stimulatory effects and dotted arrows denote inhibitory effects between inflammatory mediators. Red arrows indicate the effects of *n*-3 PUFA to increase or decrease inflammatory mediator levels, thereby subsequently up-regulating (adiponectin, *n*-3 PUFA-derived eicosanoids) or down-regulating (leptin, *n*-6 PUFA-derived eicosanoids, cytokines (TNFα, IL-1β, IL-6 and MCP-1) and macrophage tissue infiltration). TAM, tumor associated-macrophage; CLS, crown-like structure; HIF-1α, hypoxia induced factor-1α; COX-2, cyclooxygenase-2; PGE_2_, prostaglandin E_2_; TNFα, tumor necrosis factor-α; IL, interleukin; MCP-1, monocyte chemoattractant protein-1.

Marine-derived *n*-3 PUFA have well-established anti-tumorigenic effects in chemically induced, transgenic and xenograft rodent models of BC [73]. As a point of reference, amongst high LC *n*-3 PUFA consuming populations, the typical Japanese diet contains 1%–2% of daily energy as LC *n*-3 PUFA [74,75], whereas intake levels are higher amongst the Greenland Inuit who typically consume 2.4%–6.3% of daily energy as LC *n*-3 PUFA [76,77]. Although higher levels of *n*-3 PUFA intake can be achieved through supplementation, these physiologically relevant intake levels have been recapitulated in BC rodent dietary intervention studies which demonstrate a beneficial effect of *n*-3 PUFA on the BC phenotype [42,43,44,46,48,49,55,78]. In this connection, *n*-3 PUFA are recognized for their potential application in reducing obesity-associated inflammation and consequent tumorigenic risk [45]. In brief, LC *n*-3 PUFA are incorporated into mammary AT and tumor tissue [46,47], thereby increasing the levels of *n*-3 PUFA-derived lipid mediators at the expense of those derived from *n*-6 PUFA (*i.e.*, arachidonic acid (AA, C20:4*n*-6)-derived eicosanoids) [42,56,78], altering adipokine secretion [54] and interrupting tumorigenic signaling pathways [79]. These chemoprotective effects of *n*-3 PUFA result in decreased cell proliferation and increased apoptosis, ultimately resulting in reduced BC tumor incidence, growth, multiplicity, and metastasis in rodent models of BC [43,44,46,48,49,50,51,52,53,55,79]. Further, in a model of obese postmenopausal BC, *n*-3 PUFA supplementation reduced mammary AT inflammation and markers of inflammatory M1 macrophage infiltration [80] which was associated with reduced tumor burden, indicating that the inflammatory microenvironment promotes tumorigenesis and that *n*-3 PUFA directly antagonize this process. Similar *n*-3 PUFA-mediated anti-tumorigenic effects have been reported in overweight humans wherein *n*-3 PUFA supplementation up-regulated the expression of several genes involved in cell cycle regulation [81]. These studies clearly demonstrate that *n*-3 PUFA can independently modulate responsiveness to cell proliferative and/or apoptotic signaling. This is further highlighted in Figure 1, which outlines the effects of *n*-3 PUFA on critical adipokine/inflammatory mediator levels that underlie the paracrine interactions within the obese mammary tumor microenvironment that ultimately impact proliferative and apoptotic signaling and will be discussed in this review.

A recent meta-analysis that included 21 independent prospective cohort studies determined that marine source *n*-3 PUFA intake was associated with a 14% reduction in BC risk (RR = 0.86 for highest *versus* lowest category of intake (95% confidence interval 0.78–0.94)) [82]. The results were also sustained if the data was analyzed based on either reported dietary intake levels or tissue biomarker levels of *n*-3 PUFA, thereby removing concerns regarding intake compliance or accuracy in dietary recall data. In obese women, a decreased risk of BC was found to be significantly associated with both increased intake of *n*-3 PUFA and altered dietary fatty acid composition by increasing the ratio of *n*-3:*n*-6 PUFA intake, although this association was not significant in overweight or normal weight women [83]. These data suggest that obesity status may affect the association between *n*-3 PUFA intake and BC risk. To the best of our knowledge, this is the only case-control study that has specifically investigated the relationship between obesity, *n*-3 PUFA intake and BC risk. Interestingly, the chemoprotective effect of an increased ratio of *n*-3:*n*-6 PUFA intake has been reported elsewhere [84], and several case-control studies have reported an inverse association between BC risk and increased *n*-3 PUFA intake and/or increasing the *n*-3:*n*-6 PUFA ratio in breast AT [85,86,87,88]. Although obesity status was not independently assessed, some of these studies report a trend with more normal weight women in the control group and more overweight and obese women with BC [85,88]. In contrast, others have reported no association between AT *n*-3 PUFA levels and the development of BC; however, BMI was lower among these BC cases [89]. Taken together, while higher *n*-3 PUFA intake and tissue content is more often than not associated with a decreased risk of developing BC, controversy exists surrounding the associations between BC incidence, obesity status and *n*-3 PUFA intake, warranting case-control studies that investigate a relationship between all endpoints.

## 5. *n*-3 PUFA, Lipid Rafts and Breast Cancer

Extensive research has indicated that *n*-3 PUFA have a unique ability to broadly affect cell signaling. A ubiquitous mechanism by which *n*-3 PUFA can alter signal transduction is by modifying lipid rafts, which are heterogeneous, highly ordered membrane microdomains that facilitate several signaling events [90,91]. Lipid rafts laterally isolate their components from the bulk membrane and then are able to coalesce in response to stimuli to form signaling platforms [92,93], thereby playing an integral role in the propagation of multiple signaling events that are involved in tumor-promoting activities, including cell proliferation, survival, migration, and invasion [94]. The physical properties that facilitate the segregation of lipid raft domains from the bulk membrane is imparted by the enrichment of cholesterol, sphingolipids, and other phospholipids with saturated, long hydrocarbon chains within lipid rafts [95,96]. *n*-3 PUFA display low affinity for cholesterol due to their high degree of unsaturation [97,98]; therefore, enrichment of the membrane with *n*-3 PUFA can alter the composition and organization of raft domains. Altering the properties of lipid rafts can then have major effects on signaling events that are initiated or propagated by these integral domains.

An important property of lipid rafts is their size and number. Changes to the size of rafts can impose substantial changes on their function. It has been shown that rafts must be small and mobile for optimal activity [99]. Studies in BC cells, in addition to many other cell types, have demonstrated the ability of *n*-3 PUFA to alter the size of lipid rafts. Specifically, DHA was found to alter the size of lipid rafts in BC cells, resulting in lipid rafts of varying height [100]. The same study illustrated that DHA decreased the total amount of lipid rafts on the order of 20%–30%. This is particularly interesting because the levels of lipid rafts are elevated in some forms of cancer, including BC [101], and perturbing these domains can sensitize cells to apoptosis [102]. In addition to altering the size and number of lipid rafts, DHA was found to reduce cell surface levels of lipid rafts by enhancing their internalization [102]. These data implicate *n*-3 PUFA-induced changes in the physical properties of lipid rafts as a mechanism by which these fatty acids exert a chemoprotective effect.

The composition of raft domains is also central to their function as signaling platforms, and *n*-3 PUFA can substantially alter the contents of lipid rafts. The lipid composition of rafts endows the properties necessary for ordering and segregation. BC cells treated with a combination of EPA and DHA were demonstrated to have significantly reduced cholesterol, sphingomyelin, and diacylglycerol lipid raft content [103]. Another study demonstrated differential effects of EPA and DHA on the lipid composition of rafts [100]. EPA was shown to displace AA from raft domains, whereas DHA reduced cholesterol and sphingomyelin content. In addition to lipids, many proteins reside in lipid rafts and require localization to lipid rafts for signal transduction. Importantly, many of these proteins are established mediators of oncogenesis, and displacing these proteins can markedly reduce their signaling capacity. In MDA-MB-231 BC cells, several raft-associated proteins, including EGFR, Hsp90, Akt, and Src, are redistributed out of raft domains in response to DHA treatment [102], which induced increased BC cell apoptosis. Additionally, DHA was demonstrated to disrupt lipid rafts and reduce HER-2 signaling in mammary epithelial cells overexpressing HER-2 [104]. All of these proteins are involved in the regulation of cell survival and proliferation, and many are targets for cancer therapy. Another well-known therapeutic target for BC metastasis is the chemokine receptor, CXCR4. Treatment of BC cells with DHA or EPA caused redistribution of CXCR4 from lipid rafts to the cell surface [105], resulting in an overall reduction in cell migration. In addition to shifting proteins out of lipid rafts, *n*-3 PUFA can prompt the localization of some proteins into these domains. For example, CD95 (APO-1/FAS) is the transmembrane death receptor which activates the extrinsic apoptosis pathway and activation results in CD95 aggregation in the plasma membrane, followed closely by recruitment of Fas-associated death domain-containing protein (FADD) and caspase-8 to the CD95 receptor, forming the death-inducing signaling complex (DISC) [106]. EPA and DHA have been shown to induce translocation of CD95 into lipid rafts in MDA-MB-231 BC cells and this effect was accompanied by reduced cell growth [107]. Moreover, when DHA was used as a co-treatment it enhanced the chemotherapeutic effects of doxorubicin [107], an anti-neoplastic drug therapy, which has been shown to induce apoptosis and the movement of DISC to membrane rafts [108,109]. All of these data support the regulation of lipid rafts by *n*-3 PUFA as a mechanism by which they exert protective effects in BC and may also have utility as a complementary therapy in combination with pharmaceuticals, although further study is required.

## 6. Paracrine Interactions, Inflammatory Mediator Signaling and Breast Cancer

The majority of breast tissue is comprised of adipocytes, whereas epithelial cells account for only 10% of total breast cellular volume [110]. Mammary epithelial cells are embedded within the AT, which facilitates direct contact between epithelial cells and adjacent adipocytes and allows for direct functional interactions between AT and mammary tumor cells in a paracrine manner. Within the BC tumor microenvironment, these cellular interactions are further influenced by exposure to circulating AT adipokines [111,112]. Inflammation plays a role in the carcinogenic process and approximately 20% of all cancers originate in association with inflammation [113]. Given the chronic low grade-inflammatory state perpetuated in obesity [114,115], it is likely that obese AT-derived inflammatory mediator production could exacerbate inflammation-associated tumorigenic effects. Specifically, these mediators include *n*-6 PUFA-derived eicosanoids, and adipokines such as leptin and inflammatory cytokines (TNFα, IL-1β and IL-6) with a concomitant reduction in the anti-inflammatory adipokine, adiponectin (discussed below), which are produced in both visceral AT depots and surprisingly also within mammary AT depots, and collectively contribute to the development of a more severe BC phenotype via stimulating BC growth, invasion and metastasis. Typically in obesity-associated BC, inflammatory processes precede tumorigenesis. However, once developed, mammary tumor cells may also serve as a cellular source of inflammatory mediators and support the on-going inflammatory milieu within the mammary tumor microenvironment, thereby potentiating a feed-forward pro-tumorigenic mechanism facilitated by local inflammatory autocrine and paracrine interactions. In summary, autocrine and paracrine signaling between cell types within the mammary AT and tumor tissue are thought to play a central role in breast tumorigenesis [110,116], indicating that dysregulation of adipokines may underlie the association between obesity and BC.

One of the main cellular sources of these inflammatory mediators, apart from adipocytes, is macrophages that infiltrate obese AT and form crown-like structures (CLS) [117,118]. CLS are inflammatory lesions defined as adipocytes surrounded by an aggregation of macrophages that undergo necrosis and fuse to form a syncytium of lipid-containing giant multinucleated cells [119]. Obese mice have increased CLS formation in both visceral AT and mammary AT, which is associated with increased local inflammatory cytokine production (TNFα, IL-1β and IL-6), COX-2 induction and eicosanoid (PGE_2_) production, as well as increased aromatase gene expression, enzyme activity and subsequent estrogen synthesis [119,120,121,122]. Therefore, CLS formation represents a tissue localization wherein largely adipocyte-macrophage-mediated paracrine interactions promote both the development and persistence of an inflammatory AT microenvironment, which through further paracrine interactions signal to the mammary epithelial cells to promote BC growth and invasion [120,121]. Transformed mammary epithelial cells and/or the BC tumor itself can further serve as a cellular source for inflammatory mediator production and amplify the on-going local production of inflammatory mediators, and spread these signals to the surrounding non-involved mammary tissue. Moreover, in obesity, mammary AT, which is a subcutaneous AT depot, is transformed to mimic the inflammatory milieu that characterizes the obese visceral AT phenotype [120,121]. The obesity-associated mammary AT phenotypic switch is significant because evidence suggests that subcutaneous AT tends to be less inflammatory compared to visceral sources [118,123] and therefore, in obese mammary AT, a typically less inflammatory depot exhibits a more pronounced inflammatory phenotype that can drive tumorigenesis. In obesity, dietary *n*-3 PUFA supplementation has been shown to reduce AT CLS formation, reduce macrophage AT infiltration by reducing MCP-1 tissue expression and improve the inflammatory secretory profile, in part, by increasing adiponectin [27,28].

The mammary AT tumor microenvironment is complex. Increased local production of inflammatory adipokines underpin the paracrine signaling that regulates the cellular interactions between adipocytes, stromal epithelial cells and infiltrating macrophages, which ultimately drive and define mammary tumor development and the end-stage phenotype (tumor size, type and inflammatory status). Up-regulated paracrine interactions (*i.e.*, cross-talk) in obesity-associated BC perpetuate the carcinogenic process by stimulating multiple, overlapping signaling pathways. These pathways converge to stimulate aromatase expression/activation that aberrantly produces local estrogen, promote cell proliferation and/or inhibit apoptosis and stimulate the production of additional inflammatory mediators within mammary tissue, all of which ultimately support tumorigenesis. The critical inflammatory mediators that are up-regulated in obesity-associated BC perpetuate the carcinogenic process and exhibit redundant effects by stimulating multiple and overlapping signaling pathways that converge to stimulate aromatase expression/activation, resulting in aberrant local estrogen production, which promotes cell proliferation and/or inhibits apoptosis, and stimulate the production of additional inflammatory mediators within mammary tissue, ultimately resulting in tumorigenesis. Since a large and diverse list of hormones, adipokines and lipid mediators are implicated in promoting obesity-associated mammary tumorigenesis, our review will focus on a critical subset that work in concert to promote estrogen production (via aromatase activation) and tumorigenesis, specifically eicosanoids, inflammatory cytokines, leptin and adiponectin; however, we recognize that other mediators play a role in this process such as insulin, insulin-like growth factors, resistin nampt/visfatin and cholesterol as reviewed elsewhere [124,125,126]. The specific mechanisms/pathways through which obesity-associated inflammatory adipokines exert pro-tumorigenic effects are discussed in detail below, and the complexity of these paracrine interactions are shown in Figure 1.

## 7. The Role of Estrogen and Aromatase Activation in BC

Circulating estrogen levels are higher in obese women compared to lean women, and increased circulating estrogen is associated with approximately a two-fold increased risk of postmenopausal BC [5,6,127,128,129]. Additionally, obesity, particularly abdominal adiposity, increases estradiol production and bioavailability due to a reduction in hepatic synthesis of sex hormone-binding globulin (SHBG) in postmenopausal women [6,12,127,129,130]. These hormonal changes are widely believed to play an underlying role in the increased risk of BC in obese postmenopausal women [6,12,127,131]. After menopause, the primary source of estrogen are extra-ovarian sites, primarily in the AT, and aberrant AT estrogen production is attributable to increased aromatase activity, which is present at higher levels in mammary tumors compared to normal mammary tissue [132,133]. Aromatase is the rate-limiting enzyme in the estrogen biosynthesis pathway [131] which catalyzes the peripheral conversion of androstenedione and testosterone to estrone and estradiol, respectively [110]. Downstream conversion of estrone to the biologically potent estradiol is catalyzed by 17β-hydroxysteroid dehydrogenase, which is also expressed within AT [131]. In obese individuals, aromatase expression is reported to be increased by two-fold compared to normal weight individuals [125]. Additionally, aromatase expression is four to five-fold higher within breast tumor tissue compared to non-involved tissues within the same breast [125]. Consequently, mammary tissue estrogen levels are reported to be 10–50 times higher compared to blood levels in postmenopausal healthy women, which has been shown to play a critical role in BC cell growth [134,135,136,137,138].

Typically, mammary tumors are located in regions of the breast with the highest aromatase expression and activity [139,140]. Furthermore, breast tissue aromatase expression is highest in the quadrant of the breast that contains the greatest proportion of adipose stromal cells, as there is little aromatase activity in mature adipocytes [141], and accordingly, aromatase expression is typically highest in the adipose stromal cells adjacent to the tumor mass [139,140,142]. Therefore, the ratio of stromal cells to adipocytes within mammary tissue may have a predictive value in potential tumor development. Moreover, mammary tumors are typically surrounded by a layer of proliferating cancer-associated fibroblasts (CAF) which have also been shown to express aromatase, thereby indicating that factors produced by the tumor may also stimulate aromatase expression in the surrounding CAF [143].

Aromatase expression and activity is strongly influenced by local inflammatory paracrine signaling within mammary tissue. For instance, malignant epithelial cells along with AT macrophages produce pro-inflammatory mediators, including the eicosanoid PGE_2_, which induce aromatase activity and stimulate estrogen production in pre-adipocytes [131,143,144]. The resultant inflammatory mammary tissue microenvironment is further propagated in the obese state, thereby creating a favorable tissue microenvironment to promote the progression of BC growth [120,121]. Further, in obese human BC tissue, aromatase expression is associated with increased tissue levels of COX-2 and PGE_2_ [121]. In BC tissue, COX-2 expression is induced by pro-inflammatory cytokines, notably TNFα, and the resultant increased PGE_2_ levels are associated with large tumor size and high proliferation rates [131,145], due to, in part, the induction of aromatase expression via activation of cAMP-PKA and PKC-mediated signaling cascades [141,146,147]. Conversely, PGE_3_, an *n*-3 PUFA-derived eicosanoid does not induce aromatase expression [148]. Aromatase expression is negatively regulated, in part, by AMP-activated protein kinase (AMPK), which also functions as a negative regulator of the Akt/mTOR signaling pathway that is frequently activated in BC [125]. Additionally, liver kinase B1 (LKB1) can function as a tumor suppressor and can regulate aromatase expression via directly phosphorylating and activating AMPK [149]. Therefore, LKB1 and AMPK both function as negative regulators of aromatase expression in BC. As many inflammatory and metabolic factors alter aromatase expression via effects on LKB1 and/or AMPK, this may provide a critical link between obesity, inflammation and aromatase expression in BC [125]. Leptin increases aromatase expression by decreasing LKB1 protein expression and phosphorylation, whereas adiponectin exerts the opposite effect by stimulating LKB1 and its activity, leading to decreased aromatase expression [150]. Additionally, PGE_2_ down-regulates the phosphorylation of AMPK and LKB1, thereby promoting aromatase expression [150]. Inflammatory cytokines such as IL-6, IL-1β and TNFα have also been shown to stimulate aromatase activity [151,152,153,154]. TNFα induces aromatase expression through two mechanisms, (i) stimulating the binding of c-fos and c-jun transcription factors to activating protein-1 (AP-1) binding site; and (ii) activation of NFκB and MAPK signaling pathways [152,153]. Obese ovariectomized rodents exhibit increased NFκB activation and inflammatory mediator production (TNF-α, IL-1β, COX-2), which is accompanied by elevated levels of aromatase expression and activity in both the mammary gland and visceral fat [120]. Collectively, these data demonstrate that inflammatory mediator signaling in the mammary tissue microenvironment is driven by autocrine/paracrine interactions that regulate critical aspects of the mammary tissue tumor phenotype including aromatase activation and local estrogen production. These inflammatory mediators can establish a positive feedback mechanism that stimulates cell proliferation and mammary tumor development.

## 8. Inflammatory and Chemopromotive Fatty Acid Derived Lipid Mediators: Differential Effects of *n*-6 *versus* *n*-3 PUFA

LC *n*-3 and *n*-6 PUFA are metabolized by the cyclooxygenase (COX), lipoxygenase (LOX) and cytochrome P450 pathways to produce eicosanoids [72]. Specifically, *n*-6 PUFA (such as AA) serves as a substrate for COX enzymes (producing two-series prostanoids such as prostaglandins (PG) and thromboxanes (TX)), LOX enzymes (producing four-series leukotrienes (LT) and hydroxyeicosatetraenoic acids (HETE), principally 5-, 12- and 15-HETE), or cytochrome P450 enzymes (producing primarily 20-HETE) [72,148,155]. The same enzymes metabolize *n*-3 PUFA to structurally different three-series prostanoids, five-series LT, and primarily 19- and 20-hydroxyeicosapentaenoic acids (HEPE) and 21- and 22-hydroxydocosahexaenoic acids (HDHE), as well as the unique lipid mediators E- and D-series resolvins and protectins [72,148,156]. Although both resolvins and protectins exert anti-inflammatory effects, resolvins can stimulate the resolution phase of inflammation to begin at an earlier point, thereby limiting the tissue exposure to inflammatory signaling [157], whereas protectins reduce inflammatory cytokine production [158]. Generally, *n*-6 PUFA-derived eicosanoids are pro-inflammatory and pro-carcinogenic, whereas *n*-3 PUFA-derived lipid mediators are less biologically active and functionally oppose the synthesis and activity *n*-6 PUFA-derived eicosanoids [72,148,159]. Excessive dietary intake of *n*-6 PUFA *versus* *n*-3 PUFA (five- to 20-fold greater amounts) results in a significantly greater proportion of eicosanoids generated from *n*-6 PUFA [72,160]. Importantly, the fatty acid profile of adipocyte, immune and tumor cell membrane phospholipids can be modified by increased intake of *n*-3 PUFA, thereby suppressing the biosynthesis of AA-derived eicosanoids in favor of EPA and DHA-derived lipid mediators [72,148,159,161].

AA has been shown to be preferentially taken up by MDA-MB-231 BC cells in comparison to EPA, especially in a pro-inflammatory microenvironment [162]. Also, COX-2 and 12-LOX enzymes are overexpressed in BC tumor tissue [131], thereby increasing production of AA-derived inflammatory eicosanoids, which have established pro-tumorigenic effects, and dominate the BC phenotype [131,163,164,165,166]. Specifically, PGE_2_, LTB_4_ and 5-, 12- and 15-HETE, have been shown to increase cell proliferation, down-regulate apoptotic pathways, and induce rapid growth and tumor metastasis, and these effects have been shown to be counteracted by *n*-3 PUFA [50,51,159,165,166,167,168,169]. Furthermore, *n*-6 PUFA-derived eicosanoid levels are elevated during obesity and have been shown to stimulate breast tumor growth, invasion, and metastasis [131], indicating that obesity perpetuates local inflammatory eicosanoid production.

EPA is the preferential substrate for LOX enzymes, and therefore, when present in comparable proportions, *n*-3 PUFA-derived eicosanoids will be produced at the expense of *n*-6 PUFA-derived eicosanoids [148]. Moreover, *n*-3 PUFA suppress COX-2 expression, which is associated with decreased mammary epithelial cell proliferation in MMTV-HER-2/neu transgenic mice [78]. Mammary tumor PGE_2_ and 12- and 15-HETE concentrations are dose-dependently reduced by increased dietary *n*-3 PUFA intake in female nude mice injected with MDA-MB-231 cells [42,170], which is associated with increased apoptotic activity and decreased breast tumor cell proliferation, growth and lung metastasis. Similar *n*-3 PUFA-mediated anti-tumorigenic effects have been associated with suppressed cell proliferation and decreased expression of Bcl-2 and other carcinogenic proteins including Ki-67, Her-2/neu and c-Myc [50,51,159,167,168,171]. Taken together, *n*-6 PUFA-derived eicosanoids provide a link between obesity-associated chronic inflammation and the development of BC, and are a potential target for dietary LC *n*-3 PUFA intervention to mitigate the pro-carcinogenic effects of inflammatory *n*-6 PUFA-derived eicosanoids within the mammary tumor microenvironment.

## 9. Role of Inflammatory Cytokines

Inflammatory cytokines (TNFα, IL-1β, IL-6 and MCP-1) also contribute to the local mammary tissue milieu through paracrine signaling and through an autocrine positive-feedback mechanism to further their own on-going production, in part, by activating the transcription factor NFκB. Interestingly, NFκB activation underlies many aspects of BC cell proliferation, invasion and metastasis [126,172,173]. Moreover, aberrant NFκB signaling is proposed to be one of the mechanisms through which chronic inflammation leads to cancer, as NFκB activation promotes tumorigenesis by inhibiting apoptosis (via activation of Bcl2, Bcl-xL, cFLIP and other genes) and increasing cell proliferation by regulating expression of cyclinD1, cyclinE, CDK2, and c-Myc [174]. Several inflammatory mediators are up-regulated by NFκB activation; specifically, inflammatory *n*-6 PUFA-derived eicosanoid production is stimulated by NFκB activation of COX-2 [175]. Additionally, within chronically inflamed rodent mammary tissue, NFκB increases production of TNFα and IL-1β [120] and similar findings are reported in the mammary tissue of both pre- and postmenopausal obese women [121]. IL-1β levels are increased in patients with invasive ductal carcinoma and ductal carcinomas *in situ* compared to benign mammary tissue levels [176,177], and are overexpressed in breast carcinomas, but undetectable in normal breast tissue [178]. IL-1β levels are positively correlated with the expression of angiogenic factor expression, tumor grade and the expression of AP-1 [176,177,178].

IL-1β and IL-6 have been shown to stimulate BC cell proliferation in an additive manner with estrogen [154], indicative of synergy between inflammatory mediators and hormones within the mammary tumor microenvironment. TNFα, another potent inflammatory cytokine, also promotes mammary tumor development [179] and has been shown to contribute to BC cell epithelial-mesenchymal transition (EMT) by increasing matrix metalloproteinase (MMP)-9 expression, thereby enhancing migration and invasive capacity [180,181,182]. Additionally, the high levels of IL-6 and TNFα found in obese rodent adipocyte-conditioned media and serum have been shown to promote cancer cell EMT [183]. Interestingly, the two main cellular sources of TNFα are tumor-associated macrophages (TAM) and the BC cells themselves [131], highlighting the role of inflammatory macrophages in BC.

Macrophage infiltration into mammary tumor sites (and subsequent development of CLS) is driven, in part, by chemotactic signaling. MCP-1, also referred to as CCL2, signals to increase macrophage infiltration into the inflamed mammary tissue, thereby increasing the number of deleterious TAM that accumulate in mammary tumor tissue [184,185]. The cellular sources of MCP-1 in primary breast tumor sites are tumor cells and the TAM themselves, which indicates a feed-forward mechanism wherein macrophage accumulation/tumor recruitment is perpetuated throughout the stages of tumor growth [184,185]. Overall, TAM form CLS within the mammary AT and act as the cellular source of several inflammatory mediators/cytokines that perpetuate the local inflammatory tissue microenvironment, which, through autocrine and paracrine interactions, further promotes BC development [119,120,121,122,131]. Collectively, this highlights the critical role that macrophages play in driving tumor-associated inflammatory paracrine interactions. MCP-1 tumor expression is associated with a more advanced course of tumor progression, wherein MCP-1 promotes angiogenesis by stimulating the production of angiogenic factors (such as IL-8 and VEGF) [184,185]. In obesity, circulating and AT levels of MCP-1 are increased. This chemokine provides the main chemoattractant signal that drives visceral AT macrophage infiltration and CLS formation, up-regulating local AT inflammatory mediator production and subsequently impairing glucose metabolism [58,59]. Macrophage recruitment is also stimulated by AT hypoxia. In obesity, adipocyte hypertrophy results in decreased oxygen diffusion, leading to localized tissue AT hypoxia, as evidenced by upregulation of the hypoxia master regulator, hypoxia induced factor (HIF-1α), which stimulates MCP-1 and subsequent macrophage chemotaxis [143,186,187].

Countering these effects in obesity, *n*-3 PUFA have been shown to improve the hypoxic AT microenvironment by reducing adipocyte size [27] and to decrease obesity-associated expression of HIF-1α [81,188]. Furthermore, *n*-3 PUFA reduce visceral AT MCP-1 levels [27,28,35,189,190], thereby reducing obesity-associated AT macrophage accumulation, CLS formation and AT inflammation [27,28]. We and others have shown that *n*-3 PUFA reduce inflammatory paracrine signaling between adipocytes and macrophages by decreasing NFκB activation and subsequent secretion of TNFα, MCP-1 and IL-6 [191,192]. Moreover, independent of cellular source, *n*-3 PUFA have been shown to inhibit NFκB activation by decreasing IκB phosphorylation and activation, thereby reducing production TNFα, IL-1β and IL-6 [29,35,40,41,67,191,193,194].

## 10. Role of Leptin

Leptin exerts pleiotropic effects apart from regulation of energy expenditure and food intake, including effects on immunity, inflammation, cell differentiation and proliferation [124,195], all of which have direct relevance to cancer. Classically, circulating leptin levels are proportional to the amount of body fat [196] and increased leptin levels are associated with increased risk of BC development and progression [197,198]. Leptin gene expression is detected in normal healthy breast epithelial tissue [110], consistent with its endogenous role in normal mammary gland development and lactation [199]; however, it is also capable of contributing to mammary tumorigenesis [200] and is expressed in the healthy tissue that surrounds malignant ductal lesions [201]. In primary tumors and various BC cell lines, both leptin and various isoforms of the leptin receptor (ObR) including the long signaling form ObR1 are overexpressed [197,202,203,204,205,206,207]. Recently, three single nucleotide polymorphisms in the leptin receptor gene (K109R, K656N and Q223R) were identified to be associated with increased BC risk, suggesting that tumor leptin receptor signaling can directly influence tumor growth and progression [208]. Interestingly, in BC patients high intra-tumor ObR gene expression was strongly correlated with decreased relapse-free survival [209], indicating that susceptibility to leptin signaling is strongly associated with BC disease prognosis.

Leptin signaling has been shown to exert autocrine and paracrine effects, ultimately promoting cell growth and proliferation via the activation of critical signaling pathways including those mediated by PKC, c-Jun *N*-terminal kinase (JNK), p38 MAPK, Janus Kinase2/Signal Transducer and Activator of Transcription 3 (JAK2/STAT3), PI3K/Akt/mTOR, Akt/GSK3 and MAPK/extracellular signal-related kinase 1/2 (ERK1/2) pathways [116,181,195,203,210,211,212,213]. Further, leptin increases the levels of cell cycle regulators in human MCF-7 BC cells by up-regulating the expression of cyclin dependent kinase 2 (cdk2) and cyclin D1, which advances cells from the G1 to S phase of the cell cycle [214], and induces cell proliferation in ZR-75-1 BC cells via up-regulation of cyclin D1 and c-Myc [215]. Therefore, overexpression and activation of leptin within the mammary tissue microenvironment can ultimately promote tumorigenesis. Similar to obese women, rodents with high leptin levels are more likely to develop mammary tumors [216,217]. Furthermore, in animal BC models leptin antagonism has produced successful outcomes. Specifically MMTV-TGF-α mice crossed with leptin deficient mice fail to develop tumors [218,219] and treatment with a leptin antagonist decreases the growth of murine triple-negative breast tumors [220] and 4T1 mammary cancer cell growth via reducing levels of VEGF, pSTAT3 and cyclin D1 [221].

An additional role of leptin in breast carcinogenesis is to potentiate estrogen signaling, as leptin has been shown to induce aromatase expression/activity and subsequent estrogen synthesis, thereby enhancing ERα activity in BC [210,222,223]. Leptin levels positively correlate with ER expression and BC tumor size [203,205]. Further, leptin can transactivate ERα via ERK1/2 signaling [210] and enhance ERα-dependent transcription by reducing ERα ubiquitination and degradation, even in the presence of an estrogen inhibitor [223], thereby potentiating the effects of estrogen on cell proliferation. Bi-directional influences of estrogen on leptin also exist, wherein estradiol induces leptin and ObR expression, in both AT and BC cell lines [203,224,225,226].

Another obesity-associated factor that can contribute to the increased local production of leptin and subsequent pro-tumorigenic effects is AT hypoxia. Leptin receptor expression can also be stimulated by tissue hypoxia [226] and, therefore, local tissue leptin expression is up-regulated by HIF-1α [227,228]. Conversely, *n*-3 PUFA have been shown to improve the hypoxic AT microenvironment by reducing adipocyte size [27] and to decrease obesity-associated expression of HIF-1α [81,188], thereby providing a mechanism through which local leptin signaling responsiveness could be attenuated.

Dietary *n*-3 PUFA have been shown to reduce leptin AT gene expression and/or circulating levels in obese rodents [36,37] and humans [38,39], an effect that was most prominent when combined with weight loss [39]. Decreased leptin signaling represents an additional mechanism through which *n*-3 PUFA attenuate the effects of leptin. In a rodent obesity model, *n*-3 PUFA supplementation was found to decrease leptin receptor gene expression [229], thereby decreasing leptin signaling. In this connection, leptin receptors have been shown to localize to lipid rafts and downstream proliferative effects of leptin mediated through p38 MAPK signaling is lipid raft dependent [230]. Thus, *n*-3 PUFA antagonism of lipid raft size and composition in BC [100,103], as already discussed herein, may also antagonize leptin-mediated proliferative signaling within the mammary tumor microenvironment. Therefore, these findings add to the complex interplay of autocrine and paracrine interactions that underlie the obesity-associated BC inflammatory phenotype, and suggest dietary *n*-3 PUFA as an intervention that may have utility in mitigating local mammary tissue leptin production and signaling to inhibit its pro-tumorigenic effects.

## 11. Role of Adiponectin

Adipocytes are the primary cellular source of adiponectin, which is secreted as a monomeric protein that can be oligomerized to form both low-molecular weight and high molecular weight complexes [231]. Additionally, cleavage reactions via the action of elastase, can generate globular oligomeric complexes [232] that bind with greater affinity to the adiponectin receptor 1 (AdipoR1), whereas the AdipoR2 preferentially binds full-length and multimeric adiponectin [233].

Within the context of the tumor microenvironment, adiponectin and leptin counter-regulate each other and exert opposing effects [126]. Decreased levels of adiponectin may explain, in part, the increased risk of BC in obesity. Circulating adiponectin levels are generally inversely correlated with BMI, adiposity and visceral fat mass [234,235] and the decreased adiponectin levels in obesity [236] correlate with increased BC risk [111,237,238]. Moreover, three recent independent meta-analyses of BC observational studies confirmed the correlation of higher circulating adiponectin levels with lower BC risk in postmenopausal women [239,240,241]. More specifically, an increase of 3 μg/mL of circulating adiponectin corresponded to a 5% reduction in BC risk [239]. In obese postmenopausal women, hypoadiponectinemia is associated with increased BC risk, and the disease has been shown to manifest with an aggressive metastatic phenotype [235,242]. In premenopausal women, however, adiponectin levels are not associated with BC risk (95% CI −0.164 to 0.204, *p* = 0.829) [239,240,241]. The local breast tumor tissue mRNA and protein expression of adiponectin is low, although its receptors are still expressed, indicating that adiponectin-mediated anti-tumorigenic signaling is possible in BC [111,243,244]. In animal studies, reduced production of adiponectin is associated with earlier tumor onset and accelerated tumor growth [245], and overexpression of adiponectin results in mice with reduced mammary tumor size and weight [246]. Studies using various BC cell lines demonstrate that the anti-proliferative effect of adiponectin is mediated through AdipoR1 and AdipoR2 signaling [246,247,248]. There is a negative correlation between AdipoR1 expression and tumor size, which suggests that the loss of AdipoR1 signaling favours tumor growth [249]. These data are indicative of a weak autocrine/paracrine activity of this hormone within the tumor microenvironment and a loss of the beneficial anti-tumor effects of adiponectin. Despite reduced tissue levels and blunted adiponectin signaling in obesity-associated BC, treatment strategies designed to stimulate adiponectin signaling might represent a novel therapeutic approach.

Adiponectin exerts an anti-proliferative effect in BC [246,247,250,251,252,253] by impacting several signaling pathways. Specifically, adiponectin has been shown to impact the glycogen synthase kinase-3β (GSK-3β)/β-catenin signaling pathway via inhibition of phosphorylation of Akt and GSK-3β and subsequent suppression of intracellular accumulation of β-catenin and its transcriptional activities, resulting in reduced cyclin-D1 expression [246,247,251]. Additionally, adiponectin has been shown to reduce BC cell proliferation by regulating the PTEN/PI3K/mTOR and MAPK pathways [212], specifically inactivating ERK1/2, stimulating AMPK activity and decreasing Akt phosphorylation, leading to reduced mTOR activity [4,251,252,253]. In MCF-7 BC cells, microarray analysis demonstrated that adiponectin represses expression of multiple important genes that regulate cell cycle (MAPK3 and ATM) and apoptosis (BAG1, BAG3, and TP53), as well as potential diagnostic/prognostic markers (ACADS, CYP19A1, DEGS1, and EVL) [250]. Adiponectin has also been shown to induce BC cell apoptosis [251,253] by down-regulating Bcl2 and up-regulating p53, Bax and p21 expression [4,248,251]; however, this outcome is dependent on the BC cell line utilized and duration of adiponectin incubation (reviewed [212]). Adiponectin also exerts anti-inflammatory effects by inhibiting the effects of leptin [126] and inhibiting TNFα production by macrophages and adipocytes [254]. Generally, independent of cell type, adiponectin signaling down-regulates the activation of NFκB and production of inflammatory cytokines (TNFα, IL-1β, IL-6 and MCP-1) [255]. Moreover, adiponectin reduces macrophage-mediated inflammation within the tumor microenvironment by suppressing IL-6 gene expression and antagonizing NFκB, JNK and p38 MAPK mediated signaling [255,256].

Increasing the production of adiponectin in obesity may be a beneficial strategy to mitigate inflammation. *n*-3 PUFA have been shown to up-regulate adiponectin secretion in both murine [65] and human adipocytes [66]. Furthermore, dietary *n*-3 PUFA improve the obesity-associated inflammatory secretory profile, in part, by increasing adiponectin levels [27,28,29,30,31,35,63]. In human clinical trials, dietary *n*-3 PUFA have been shown to increase adiponectin levels [32,33,34] in obese and overweight subjects, thereby demonstrating the potential utility of *n*-3 PUFA to stimulate the effects of this anti-inflammatory adipokine in obesity. Considering the anti-tumorigenic effects of adiponectin and the ability of *n*-3 PUFA to restore adiponectin function in obesity [27,28,29,30,31,32,33,34,35,63], further research initiatives should be undertaken to determine the utility of *n*-3 PUFA in mitigating obesity-associated BC inflammation and tumor production.

## 12. Conclusions

In obese women with BC, increased inflammatory adipokine production, both locally in the mammary AT depot and systemically, perpetuates inflammation-associated pro-tumorigenic signaling pathways, thereby increasing disease severity. A spectrum of inflammatory mediators/adipokines are produced by adipocytes, TAM, mammary epithelial cells and tumor cells, which collectively stimulate diverse and overlapping signaling pathways that converge to stimulate aromatase activity that aberrantly increases local estrogen production, up-regulates cell proliferation and down-regulates apoptosis. The complex nature of the obesity-associated BC inflammatory pathophysiology is not likely to be attenuated or prevented by targeting any individual inflammatory mediator and/or signaling pathway, which may explain why most drug therapies, in this context, are ineffective. Instead, a pan-anti-inflammatory approach is more likely to have success in mitigating obesity-associated mammary tissue inflammatory paracrine interactions and subsequent tumorigenesis, and in this context, *n*-3 PUFA may have utility. *n*-3 PUFA have been shown to concurrently target multiple aspects of the obese BC phenotype including reduction of macrophage AT infiltration and CLS formation and down-regulation of critical adipokine production. Collectively, increased *n*-3 PUFA intake could attenuate obesity-associated BC. Considering the current state of obesity world-wide, further studies in human at risk populations should be made a priority.
